# 2,2′-Bipyridin-1-ium 3-nitro­benzene­sulfonate

**DOI:** 10.1107/S1600536810003909

**Published:** 2010-02-06

**Authors:** Ersin Temel, Beratiye Tokgöz, Turan Kaya Yazıcılar, Orhan Büyükgüngör

**Affiliations:** a Department of Physics, Faculty of Arts and Sciences, Ondokuz Mayıs University, Kurupelit, TR-55139 Samsun, Turkey; b Department of Chemistry, Faculty of Arts and Sciences, Ondokuz Mayıs University, Kurupelit, TR-55139 Samsun, Turkey

## Abstract

In the title compound, C_10_H_9_N_2_^+^·C_6_H_4_NO_5_S^−^, the dihedral angle between the aromatic rings of the cation is 9.42 (7)°. In the crystal, the anions and cations are linked by C—H⋯O and N—H⋯O hydrogen bonds, generating *R*_2_^1^(5) and *R*_4_^4^(14) rings, respectively. These hydrogen bonds also provide packing along [110].

## Related literature

For 2,2′-bipyridinium, see: Grummt *et al.* (2004 ▶); Branowska *et al.* (2005 ▶); Zhang *et al.* (2007 ▶); Kavitha *et al.* (2006 ▶). For aromatic sulfonates, see: Sharma *et al.* (2004 ▶); Vembu *et al.* (2009 ▶). For graph-set notation, see: Bernstein *et al.* (1995 ▶).
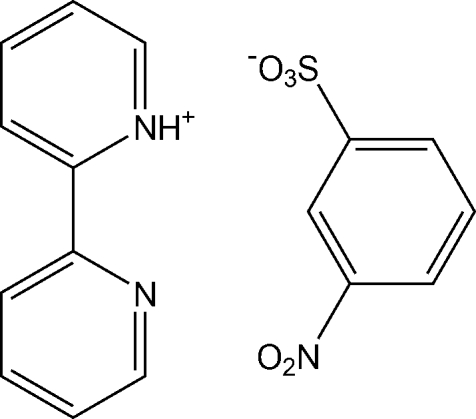


## Experimental

### 

#### Crystal data

C_10_H_9_N_2_^+^·C_6_H_4_NO_5_S^−^
*M* *_r_* = 359.36
Triclinic, 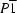

*a* = 5.9527 (3) Å
*b* = 7.4674 (3) Å
*c* = 17.8800 (7) Å
α = 79.085 (3)°
β = 89.121 (4)°
γ = 87.939 (3)°
*V* = 779.87 (6) Å^3^
*Z* = 2
Mo *K*α radiation
μ = 0.24 mm^−1^
*T* = 296 K
0.60 × 0.51 × 0.38 mm

#### Data collection

Stoe IPDS II diffractometer
Absorption correction: integration (*X-RED32*, Stoe & Cie, 2002 ▶) *T*_min_ = 0.859, *T*_max_ = 0.935
17821 measured reflections
3069 independent reflections
2997 reflections with *I* > 2σ(*I*)
*R*_int_ = 0.042

#### Refinement

*R*[*F* ^2^ > 2σ(*F* ^2^)] = 0.033
*wR*(*F* ^2^) = 0.088
*S* = 1.05
3069 reflections
230 parameters
H atoms treated by a mixture of independent and constrained refinement
Δρ_max_ = 0.18 e Å^−3^
Δρ_min_ = −0.39 e Å^−3^

### 

Data collection: *X-AREA* (Stoe & Cie, 2002 ▶); cell refinement: *X-AREA*; data reduction: *X-RED32* (Stoe & Cie, 2002 ▶); program(s) used to solve structure: *SHELXS97* (Sheldrick, 2008 ▶); program(s) used to refine structure: *SHELXL97* (Sheldrick, 2008 ▶); molecular graphics: *ORTEP-3 for Windows* (Farrugia, 1997 ▶); software used to prepare material for publication: *WinGX* (Farrugia, 1999 ▶).

## Supplementary Material

Crystal structure: contains datablocks I, global. DOI: 10.1107/S1600536810003909/fj2270sup1.cif

Structure factors: contains datablocks I. DOI: 10.1107/S1600536810003909/fj2270Isup2.hkl

Additional supplementary materials:  crystallographic information; 3D view; checkCIF report

## Figures and Tables

**Table 1:** Hydrogen-bond geometry (Å, °)

*D*—H⋯*A*	*D*—H	H⋯*A*	*D*⋯*A*	*D*—H⋯*A*
C1—H1⋯O4^i^	0.93	2.55	3.1426 (19)	122
C2—H2⋯O4^i^	0.93	2.55	3.142 (2)	122
C4—H4⋯O5^ii^	0.93	2.45	3.3089 (19)	153
N1—H1*A*⋯O3	0.88 (2)	1.95 (2)	2.7096 (16)	142.9 (18)

Symmetry codes: (i) 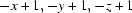; (ii) 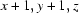.

**Fig. 1.**
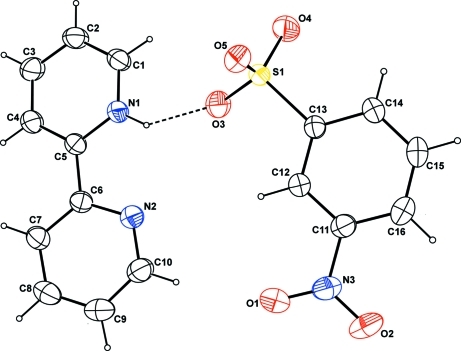
The molecular structure of the title compound, showing the atom-numbering scheme. Displacement ellipsoids are drawn at the 40% probability. Hydrogen bonds are indicated by dashed lines.

**Fig. 2.**
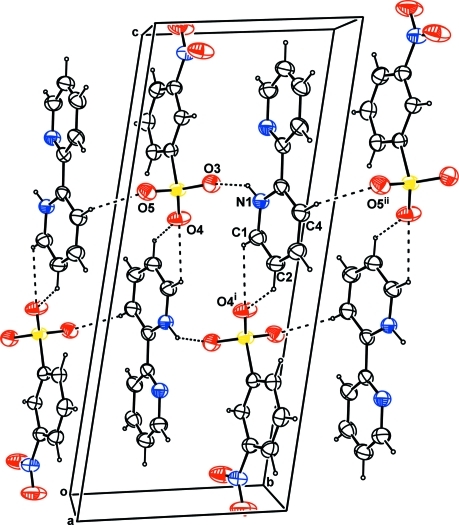
Partial packing part of the crystal structure of the title compound showing the formation of R21(5) and R44(14) rings running through the direction [110] generated by C—H···O and N—H..O type hydrogen bonds. Symmetry codes: (i) 1 - x, 1 - y, 1 - z; (ii) 1 + x, 1 + y, z.

## supplementary crystallographic information

### Comment

In this study, we have examined the title compound obtained by synthesize of the *meta*-nitro benzene sulfonic acid sodium salt and the bipyridine. The 2,2'-bipyridine and its fused analogues are exhibit important application in coordination and supramolecular chemistry (Grummt *et al.*, 2004; Branowska *et al.*, 2005; Zhang *et al.*, 2007; Kavitha *et al.*, 2006) while aromatic sulfonates draw attention because of their industrial applications as surfactants, dyes, fuel and lubricant detergents (Sharma *et al.*, 2004; Vembu *et al.*, 2009).

The asymmetric unit of the title compound contains protonated 2,2'-bipyridin-1-ium cation and deprotonated *m*-nitrobenzenesulfonate anion. A perspective unit of the asymmetric unit is presented in Fig. 1. In the asymmetric unit, the mean planes of the anionic (except O atoms bounded S atom) and cationic moieties oriented to each other with 6.88 (5)°. However, the protonated and deprotonated rings of bipyridinium oriented to the aromatic ring of anionic moiety with 9.16 (5)° and 4.79 (5)°, respectively. The two pyridine rings forming bipyridinium moiety are slightly twisted by 9.42 (7)°.

For the bipyridinium cation, the N—C bond distances are in the range 1.331 (2) Å-1.3446 (18)Å and, as expected, the protonated part of the bipyridinium has slightly different bond lengths and angles than non-protonated part. The C1—N1—C5 angle [123.75 (13)°] is larger than the C6—N2—C10 angle [117.10 (14)°].

The S—O bond distances for *m*-nitrobenzenesulfonate are in the range 1.4370 (13) Å-1.4466 (13) Å, while the adjacent O—S—O angles are in the range 112.77 (8)–113.72 (8)°. The nitro group is slightly deviated from planarity with the dihedral angle of 2.98 (16)° between the benzene and nitro moieties.

The anionic and cationic moieties are linked by C—H···O and N—H···O type hydrogen bonds. In the structure, the nitrobenzene sulfonate anion acts as donor while bipyridinium cation acts as acceptor. The C1—H1···O4^i^ and C2—H2···O4^i^ interactions constitute a bifurcated acceptor bonds generating *R*_2_^1^(5) rings in graph-set notation (Bernstein *et al.*, 1995). It is also found that the *R*_4_^4^(14) ring motives are generated by the N1—H1A···O3 and C1—H1···O4^i^ hydrogen bonds (Fig. 2). These adjacent rings are provide packing along to the direction [110]. Furthermore, the intra-molecular N1—H1A···N2 hydrogen bonds generate S(5) ring motives (Fig. 1). Geometric details of hydrogen bonds are given in Table 1.

### Experimental

1.944 g Mn-BSA (3 mmol) was dissolved in 10 ml of water and a solution of bipyridine (0.937 g, 6 mmol) in 3 ml e thanol was added into the solution. After stirring for 10 minutes, the solution was left for crystallization and a precipitate of Mn(OH)_2_ was obtained and resolved again with the addition of 2 ml concentrate HCI into the mixture. Two different forms of crystals, one has a yellow colour while other is colurless, were grown after one day. The crystals were washed with slightly acidic water to separate from each other. The yellow one was resolved whereas the colourless one was not within the acidic solution. Transparent square-shaped crystals of X-ray quality were dried *in vacuo*.

### Refinement

The H atom bonded to N atom was located in Fourier map and refined isotropically. Other H atoms were placed in calculated positions, with C—H = 0.93 Å, and refined in riding model, with *U*_iso_(H) = 1.2*U*_eq_(C).

### Crystal data

**Table:**

C_10_H_9_N_2_^+^·C_6_H_4_NO_5_S^−^	*Z* = 2
*M**_r_* = 359.36	*F*(000) = 372
Triclinic, *P*1	*D*_x_ = 1.529 Mg m^−^^3^
Hall symbol: -P 1	Mo *K*α radiation, λ = 0.71073 Å
*a* = 5.9527 (3) Å	Cell parameters from 3236 reflections
*b* = 7.4674 (3) Å	θ = 2.3–28.1°
*c* = 17.8800 (7) Å	µ = 0.24 mm^−^^1^
α = 79.085 (3)°	*T* = 296 K
β = 89.121 (4)°	Prism, colourless
γ = 87.939 (3)°	0.60 × 0.51 × 0.38 mm
*V* = 779.87 (6) Å^3^	

### Data collection

**Table:**

Stoe IPDS II diffractometer	3069 independent reflections
Radiation source: fine-focus sealed tube	2997 reflections with *I* > 2σ(*I*)
plane graphite	*R*_int_ = 0.042
Detector resolution: 6.67 pixels mm^-1^	θ_max_ = 26.0°, θ_min_ = 2.3°
rotation method scans	*h* = −7→7
Absorption correction: integration (*X-RED32*, Stoe & Cie, 2002)	*k* = −9→9
*T*_min_ = 0.859, *T*_max_ = 0.935	*l* = −22→22
17821 measured reflections	

### Refinement

**Table:**

Refinement on *F*^2^	Primary atom site location: structure-invariant direct methods
Least-squares matrix: full	Secondary atom site location: difference Fourier map
*R*[*F*^2^ > 2σ(*F*^2^)] = 0.033	Hydrogen site location: inferred from neighbouring sites
*wR*(*F*^2^) = 0.088	H atoms treated by a mixture of independent and constrained refinement
*S* = 1.05	*w* = 1/[σ^2^(*F*_o_^2^) + (0.0399*P*)^2^ + 0.2343*P*] where *P* = (*F*_o_^2^ + 2*F*_c_^2^)/3
3069 reflections	(Δ/σ)_max_ = 0.001
230 parameters	Δρ_max_ = 0.18 e Å^−^^3^
0 restraints	Δρ_min_ = −0.39 e Å^−^^3^

### Special details

**Table:**

Geometry. All e.s.d.'s (except the e.s.d. in the dihedral angle between two l.s. planes) are estimated using the full covariance matrix. The cell e.s.d.'s are taken into account individually in the estimation of e.s.d.'s in distances, angles and torsion angles; correlations between e.s.d.'s in cell parameters are only used when they are defined by crystal symmetry. An approximate (isotropic) treatment of cell e.s.d.'s is used for estimating e.s.d.'s involving l.s. planes.
Refinement. Refinement of *F*^2^ against ALL reflections. The weighted *R*-factor *wR* and goodness of fit *S* are based on *F*^2^, conventional *R*-factors *R* are based on *F*, with *F* set to zero for negative *F*^2^. The threshold expression of *F*^2^ > σ(*F*^2^) is used only for calculating *R*-factors(gt) *etc*. and is not relevant to the choice of reflections for refinement. *R*-factors based on *F*^2^ are statistically about twice as large as those based on *F*, and *R*- factors based on ALL data will be even larger.

### Fractional atomic coordinates and isotropic or equivalent isotropic displacement parameters (Å^2^)

**Table:**

	*x*	*y*	*z*	*U*_iso_*/*U*_eq_	
C1	0.9249 (3)	0.6631 (2)	0.55700 (9)	0.0472 (3)	
H1	0.7963	0.6145	0.5410	0.057*	
C2	1.0720 (3)	0.7523 (2)	0.50459 (9)	0.0518 (4)	
H2	1.0453	0.7652	0.4527	0.062*	
C3	1.2602 (3)	0.8224 (2)	0.53019 (10)	0.0551 (4)	
H3	1.3633	0.8815	0.4953	0.066*	
C4	1.2977 (3)	0.8059 (2)	0.60717 (9)	0.0495 (4)	
H4	1.4235	0.8563	0.6241	0.059*	
C5	1.1473 (2)	0.71416 (19)	0.65879 (8)	0.0393 (3)	
C6	1.1649 (2)	0.68668 (19)	0.74248 (8)	0.0399 (3)	
C7	1.3558 (3)	0.7287 (2)	0.77801 (9)	0.0475 (4)	
H7	1.4820	0.7711	0.7497	0.057*	
C8	1.3540 (3)	0.7062 (2)	0.85644 (10)	0.0534 (4)	
H8	1.4785	0.7354	0.8820	0.064*	
C9	1.1657 (3)	0.6399 (2)	0.89627 (9)	0.0554 (4)	
H9	1.1601	0.6244	0.9491	0.066*	
C10	0.9862 (3)	0.5970 (2)	0.85640 (9)	0.0532 (4)	
H10	0.8611	0.5490	0.8838	0.064*	
C11	0.2858 (2)	0.1898 (2)	0.87745 (8)	0.0416 (3)	
C12	0.4108 (2)	0.24262 (19)	0.81181 (8)	0.0383 (3)	
H12	0.5512	0.2924	0.8135	0.046*	
C13	0.3213 (2)	0.21927 (18)	0.74351 (8)	0.0362 (3)	
C14	0.1093 (2)	0.1485 (2)	0.74167 (9)	0.0416 (3)	
H14	0.0484	0.1353	0.6955	0.050*	
C15	−0.0112 (2)	0.0977 (2)	0.80866 (10)	0.0475 (4)	
H15	−0.1527	0.0497	0.8072	0.057*	
C16	0.0758 (3)	0.1174 (2)	0.87731 (9)	0.0476 (4)	
H16	−0.0047	0.0828	0.9224	0.057*	
N1	0.9666 (2)	0.64630 (17)	0.63119 (7)	0.0415 (3)	
N2	0.9811 (2)	0.62032 (18)	0.78077 (7)	0.0467 (3)	
N3	0.3810 (3)	0.2146 (2)	0.94976 (7)	0.0536 (3)	
O1	0.5688 (2)	0.2735 (2)	0.94961 (8)	0.0795 (5)	
O2	0.2667 (3)	0.1770 (3)	1.00732 (8)	0.0897 (5)	
O3	0.6150 (2)	0.42572 (17)	0.66965 (7)	0.0617 (3)	
O4	0.3309 (2)	0.3147 (2)	0.59711 (7)	0.0657 (4)	
O5	0.62591 (19)	0.11015 (17)	0.65774 (7)	0.0547 (3)	
S1	0.48694 (6)	0.27252 (5)	0.659004 (19)	0.04088 (12)	
H1A	0.875 (3)	0.581 (3)	0.6636 (12)	0.062 (5)*	

### Atomic displacement parameters (Å^2^)

**Table:**

	*U*^11^	*U*^22^	*U*^33^	*U*^12^	*U*^13^	*U*^23^
C1	0.0479 (8)	0.0537 (9)	0.0416 (8)	−0.0113 (7)	−0.0061 (6)	−0.0109 (6)
C2	0.0611 (10)	0.0582 (9)	0.0368 (8)	−0.0129 (8)	−0.0020 (7)	−0.0086 (7)
C3	0.0602 (10)	0.0616 (10)	0.0444 (9)	−0.0224 (8)	0.0095 (7)	−0.0093 (7)
C4	0.0476 (8)	0.0567 (9)	0.0473 (8)	−0.0203 (7)	0.0024 (7)	−0.0145 (7)
C5	0.0392 (7)	0.0404 (7)	0.0405 (7)	−0.0068 (6)	−0.0010 (6)	−0.0123 (6)
C6	0.0417 (7)	0.0395 (7)	0.0406 (7)	−0.0033 (6)	−0.0011 (6)	−0.0121 (6)
C7	0.0434 (8)	0.0533 (9)	0.0489 (9)	−0.0038 (7)	−0.0046 (7)	−0.0166 (7)
C8	0.0551 (9)	0.0565 (9)	0.0522 (9)	0.0040 (7)	−0.0161 (7)	−0.0191 (7)
C9	0.0696 (11)	0.0583 (10)	0.0394 (8)	0.0060 (8)	−0.0064 (8)	−0.0130 (7)
C10	0.0582 (10)	0.0587 (10)	0.0422 (8)	−0.0048 (8)	0.0049 (7)	−0.0085 (7)
C11	0.0433 (7)	0.0448 (8)	0.0365 (7)	0.0007 (6)	0.0016 (6)	−0.0077 (6)
C12	0.0339 (7)	0.0437 (7)	0.0381 (7)	−0.0040 (6)	0.0000 (5)	−0.0089 (6)
C13	0.0337 (6)	0.0380 (7)	0.0367 (7)	−0.0052 (5)	−0.0001 (5)	−0.0060 (5)
C14	0.0347 (7)	0.0449 (8)	0.0461 (8)	−0.0053 (6)	−0.0030 (6)	−0.0096 (6)
C15	0.0332 (7)	0.0481 (8)	0.0604 (10)	−0.0075 (6)	0.0055 (6)	−0.0080 (7)
C16	0.0436 (8)	0.0489 (8)	0.0486 (8)	−0.0032 (6)	0.0121 (7)	−0.0055 (7)
N1	0.0404 (6)	0.0464 (7)	0.0389 (6)	−0.0118 (5)	0.0005 (5)	−0.0087 (5)
N2	0.0470 (7)	0.0523 (7)	0.0417 (7)	−0.0082 (6)	0.0016 (5)	−0.0103 (5)
N3	0.0599 (9)	0.0650 (9)	0.0364 (7)	−0.0018 (7)	0.0023 (6)	−0.0109 (6)
O1	0.0639 (8)	0.1318 (14)	0.0470 (7)	−0.0203 (9)	−0.0070 (6)	−0.0242 (8)
O2	0.1000 (11)	0.1333 (14)	0.0388 (7)	−0.0341 (10)	0.0193 (7)	−0.0201 (8)
O3	0.0661 (7)	0.0675 (8)	0.0542 (7)	−0.0363 (6)	0.0115 (6)	−0.0130 (6)
O4	0.0549 (7)	0.1012 (10)	0.0376 (6)	−0.0137 (7)	−0.0108 (5)	−0.0014 (6)
O5	0.0484 (6)	0.0670 (7)	0.0517 (7)	−0.0053 (5)	0.0090 (5)	−0.0189 (5)
S1	0.03790 (19)	0.0538 (2)	0.03193 (18)	−0.01426 (15)	−0.00014 (13)	−0.00822 (14)

### Geometric parameters (Å, °)

**Table:**

C1—N1	1.335 (2)	C10—H10	0.9300
C1—C2	1.367 (2)	C11—C16	1.380 (2)
C1—H1	0.9300	C11—C12	1.381 (2)
C2—C3	1.373 (2)	C11—N3	1.466 (2)
C2—H2	0.9300	C12—C13	1.383 (2)
C3—C4	1.379 (2)	C12—H12	0.9300
C3—H3	0.9300	C13—C14	1.3891 (19)
C4—C5	1.379 (2)	C13—S1	1.7798 (14)
C4—H4	0.9300	C14—C15	1.383 (2)
C5—N1	1.3446 (18)	C14—H14	0.9300
C5—C6	1.476 (2)	C15—C16	1.375 (2)
C6—N2	1.3413 (19)	C15—H15	0.9300
C6—C7	1.384 (2)	C16—H16	0.9300
C7—C8	1.380 (2)	N1—H1A	0.88 (2)
C7—H7	0.9300	N3—O1	1.2153 (19)
C8—C9	1.375 (3)	N3—O2	1.2180 (19)
C8—H8	0.9300	O3—S1	1.4406 (12)
C9—C10	1.374 (2)	O4—S1	1.4371 (12)
C9—H9	0.9300	O5—S1	1.4466 (12)
C10—N2	1.331 (2)		
			
N1—C1—C2	119.67 (14)	C16—C11—N3	119.28 (13)
N1—C1—H1	120.2	C12—C11—N3	117.98 (13)
C2—C1—H1	120.2	C11—C12—C13	118.09 (13)
C1—C2—C3	118.59 (15)	C11—C12—H12	121.0
C1—C2—H2	120.7	C13—C12—H12	121.0
C3—C2—H2	120.7	C12—C13—C14	120.33 (13)
C2—C3—C4	120.66 (15)	C12—C13—S1	118.88 (10)
C2—C3—H3	119.7	C14—C13—S1	120.75 (11)
C4—C3—H3	119.7	C15—C14—C13	119.86 (14)
C5—C4—C3	119.53 (14)	C15—C14—H14	120.1
C5—C4—H4	120.2	C13—C14—H14	120.1
C3—C4—H4	120.2	C16—C15—C14	120.79 (14)
N1—C5—C4	117.78 (14)	C16—C15—H15	119.6
N1—C5—C6	116.68 (13)	C14—C15—H15	119.6
C4—C5—C6	125.53 (13)	C15—C16—C11	118.18 (14)
N2—C6—C7	123.11 (14)	C15—C16—H16	120.9
N2—C6—C5	114.67 (13)	C11—C16—H16	120.9
C7—C6—C5	122.21 (14)	C1—N1—C5	123.75 (13)
C8—C7—C6	118.32 (15)	C1—N1—H1A	117.7 (13)
C8—C7—H7	120.8	C5—N1—H1A	118.4 (13)
C6—C7—H7	120.8	C10—N2—C6	117.10 (14)
C9—C8—C7	119.08 (15)	O1—N3—O2	122.94 (16)
C9—C8—H8	120.5	O1—N3—C11	118.79 (13)
C7—C8—H8	120.5	O2—N3—C11	118.26 (15)
C10—C9—C8	118.62 (15)	O4—S1—O3	113.69 (8)
C10—C9—H9	120.7	O4—S1—O5	113.72 (8)
C8—C9—H9	120.7	O3—S1—O5	112.77 (8)
N2—C10—C9	123.73 (16)	O4—S1—C13	106.14 (7)
N2—C10—H10	118.1	O3—S1—C13	104.51 (7)
C9—C10—H10	118.1	O5—S1—C13	104.90 (7)
C16—C11—C12	122.74 (14)		
			
N1—C1—C2—C3	0.0 (3)	C13—C14—C15—C16	0.4 (2)
C1—C2—C3—C4	−1.1 (3)	C14—C15—C16—C11	0.3 (2)
C2—C3—C4—C5	1.6 (3)	C12—C11—C16—C15	−0.1 (2)
C3—C4—C5—N1	−1.0 (2)	N3—C11—C16—C15	179.13 (14)
C3—C4—C5—C6	−179.67 (15)	C2—C1—N1—C5	0.6 (2)
N1—C5—C6—N2	−9.30 (19)	C4—C5—N1—C1	−0.1 (2)
C4—C5—C6—N2	169.42 (15)	C6—C5—N1—C1	178.68 (14)
N1—C5—C6—C7	171.48 (14)	C9—C10—N2—C6	1.2 (3)
C4—C5—C6—C7	−9.8 (2)	C7—C6—N2—C10	0.8 (2)
N2—C6—C7—C8	−2.0 (2)	C5—C6—N2—C10	−178.46 (13)
C5—C6—C7—C8	177.20 (14)	C16—C11—N3—O1	177.87 (16)
C6—C7—C8—C9	1.3 (2)	C12—C11—N3—O1	−2.9 (2)
C7—C8—C9—C10	0.5 (3)	C16—C11—N3—O2	−2.9 (2)
C8—C9—C10—N2	−1.8 (3)	C12—C11—N3—O2	176.31 (16)
C16—C11—C12—C13	−0.8 (2)	C12—C13—S1—O4	−154.95 (12)
N3—C11—C12—C13	179.98 (13)	C14—C13—S1—O4	27.62 (14)
C11—C12—C13—C14	1.5 (2)	C12—C13—S1—O3	−34.49 (13)
C11—C12—C13—S1	−175.98 (11)	C14—C13—S1—O3	148.08 (12)
C12—C13—C14—C15	−1.3 (2)	C12—C13—S1—O5	84.36 (12)
S1—C13—C14—C15	176.12 (11)	C14—C13—S1—O5	−93.07 (13)

### Hydrogen-bond geometry (Å, °)

**Table:**

*D*—H···*A*	*D*—H	H···*A*	*D*···*A*	*D*—H···*A*
C1—H1···O4^i^	0.93	2.55	3.1426 (19)	122
C2—H2···O4^i^	0.93	2.55	3.142 (2)	122
C4—H4···O5^ii^	0.93	2.45	3.3089 (19)	153
N1—H1A···O3	0.88 (2)	1.95 (2)	2.7096 (16)	142.9 (18)

Symmetry codes: (i) −*x*+1, −*y*+1, −*z*+1; (ii) *x*+1, *y*+1, *z*.
